# Diné (Navajo) female perspectives on mother–daughter communication and cultural assets around the transition to womanhood: a cross-sectional survey

**DOI:** 10.1186/s12905-021-01473-4

**Published:** 2021-09-25

**Authors:** Jennifer Richards, Rachel Strom Chambers, Jaime Lynn Begay, Kendrea Jackson, Lauren Tingey, Hima Patel, Scott Carvajal, Stephanie Russo Carroll, Nicolette Teufel-Shone, Allison Barlow

**Affiliations:** 1 Center for American Indian Health, Johns Hopkins Bloomberg School of Public Health, Tuba City, AZ 86045 USA; 2 Center for American Indian Health, Johns Hopkins Bloomberg School of Public Health, Baltimore, MD 21231 USA; 3 Center for American Indian Health, Johns Hopkins Bloomberg School of Public Health, Chinle, AZ 86503 USA; 4 Center for American Indian Health, Bloomberg School of Public Health, Johns Hopkins University, Baltimore, USA; 5 Center for American Indian Health, Johns Hopkins Bloomberg School of Public Health, Baltimore, USA; 6 Health Behavior Health Promotion, Mel and Enid Zuckerman College of Public Health, University of Arizona, Tucson, AZ 85724 USA; 7 Public Health Policy and Management, Mel and Enid Zuckerman College of Public Health, University of Arizona, Tucson, AZ 85724 USA; 8 Health Sciences, Northern Arizona University, Flagstaff, AZ 86011 USA

**Keywords:** Mother–daughter, American Indian, Indigenous, Reproductive health, Culturally grounded curricula, Preconception

## Abstract

**Background:**

The inclusion of protective factors (“assets”) are increasingly supported in developing culturally grounded interventions for American Indian (AI) populations. This study sought to explore AI women’s cultural assets, perspectives, and teachings to inform the development of a culturally grounded, intergenerational intervention to prevent substance abuse and teenage pregnancy among AI females.

**Methods:**

Adult self-identified AI women (N = 201) who reside on the Navajo Nation completed a cross-sectional survey between May and October 2018. The 21-question survey explored health communication around the transition to womanhood, cultural assets, perceptions of mother–daughter reproductive health communication, and intervention health topics. Univariate descriptive analyses, chi squared, and fisher’s exact tests were conducted.

**Results:**

Respondents ranged in age from 18 to 82 years, with a mean age of 44 ± 15.5 years. Women self-identified as mothers (95; 48%), aunts (59; 30%), older sisters (55; 28%), grandmothers (37; 19%), and/or all of the aforementioned (50; 25%). 66% (N = 95) of women admired their mother/grandmother most during puberty; 29% (N = 58) of women were 10–11 years old when someone first spoke to them about menarche; and 86% (N=172) felt their culture was a source of strength. 70% (N = 139) would have liked to learn more about reproductive health when they were a teenager; 67% (N = 134) felt Diné mothers are able to provide reproductive health education; 51% (N = 101) reported having a rite of passage event, with younger women desiring an event significantly more than older women. Responses also indicate a disruption of cultural practices due to government assimilation policies, as well as the support of male relatives during puberty.

**Conclusions:**

Results informed intervention content and delivery, including target age group, expanded caregiver eligibility criteria, lesson delivery structure and format, and protective cultural teachings. Other implications include the development of a complementary fatherhood and/or family-based intervention to prevent Native girls’ substance use and teen pregnancy.

**Supplementary Information:**

The online version contains supplementary material available at 10.1186/s12905-021-01473-4.

## Background

The dual challenge of early substance use and teenage pregnancy are two of the most critical areas of concern for American Indian and Alaska Native (AIAN) communities [1–3]. Adolescent substance use, in particular, has the potential to initiate adverse consequences throughout the life course in addition to unintended teen pregnancy, including: violence, injuries, sexually transmitted infections (STIs), physical or sexual assault, impaired adolescent brain development, suicide, and school dropout [1, 4–9]. Especially alarming is that AIAN youth initiate substances earlier than non-AI youth [6, 10–12].

The impact of substance use is compounded for AIAN females as studies have shown that early substance use is closely linked to sexual risk taking and teen pregnancy among adolescent girls [1, 13–17]. It is not surprising then that AIAN females have higher STI (age 15–24 years) and teenage pregnancy rates than any other U.S. females racial/ethnic group [1, 18–23]. Walls et al. found that Southwest AI girls reported significantly more drug offers and difficulty in drug refusal than their male counterparts [24]. This is concerning because studies indicate teenage girls are also at higher risk for substance dependence [25]. Such findings elucidate an increasing awareness of AI gender-specific factors that drive high-risk behaviors, including substance use [8, 12, 26, 27]. For example, boys more often report they use substances for sensation-seeking while girls use substances to boost their confidence, cope with stress, or control their weight [28].

Mother-daughter dyadic strategies are specifically supported because of their bilateral health influence and potential for sustained behavior change [29–33]. Engaging mothers as the primary health educators of early adolescents is a promising strategy to reducing adolescent girls’ early substance use and sexual behaviors [30, 32, 34–38]. Despite the increasing support for adolescent mother-daughter interventions, there is a minimal literature on this dyadic strategy in Native communities [33, 39, 40]. However, previous studies support family-, parent-, and cultural-connectedness as buffers against high risk behaviors among AI youth and provide rationale to explore intergenerational approaches to substance abuse and teen pregnancy prevention [41–44].

The Native female adult/child relationship is an especially unique and unexplored locus of behavior change. Many tribes extend maternal sources of support to include grandmothers, aunts, cousins, and female relatives [45–47]. Extensive female kinship networks, reinforced through cultural puberty ceremonies, provide reason to include older female relatives in mother-daughter interventions and to identify who communicates reproductive health education to Native girls [45, 48, 49].

The Diné (Navajo), well-known as a matrilineal society, centers familial, community, kinship, and land resources around female intergenerational relationships [49, 50]. Enforced from all tiers of Diné society, there is reason to explore how programs can leverage these intergenerational relationships to improve the health of young girls and prevent substance use and teen pregnancy. This manuscript presents findings from a questionnaire exploring the protective cultural assets present among Diné women across generations, including health communication around the transition to womanhood, cultural assets during the transition to womanhood, perceptions of mother-daughter reproductive health communication and mother-daughter intervention topics. The questionnaire is part of a formative community-based participatory research project at the Johns Hopkins Center for American Indian Health (JHCAIH), which includes focus groups, in-depth interviews/storytelling, and cultural consultation. Findings from formative activities, including this questionnaire, will inform the development of a mother-daughter intervention aimed at promoting reproductive health and preventing substance use and teen pregnancy among Diné girls.

## Methods

American Indian (AI) women over the age of 18 years who self-identified as female and resided on the Navajo Nation were the participants in this study. With over 330,000 members nationwide, the Navajo Nation, where this study takes place is the second most populated tribal nation [51]. Approximately 157,000 (47%) tribal members reside on the Navajo Nation, which spans nearly 27,500 square miles and stretches into Arizona, New Mexico, and Utah [51]. The populations of the two communities involved in this study are 9300 and 8000, respectively [51]. Since the intent of the survey was not to establish causal relationships or to achieve external validity, a sample size of 200 AI women was considered feasible and appropriate. JHCAIH staff aimed to recruit 100 AI women per site at high-traffic community events such as flea markets, grocery stores, and health fairs. This study and the survey were approved by the Johns Hopkins University Institutional Review Board and the Navajo Nation Human Research Review Board. Local and regional tribal government approvals were also obtained for participating communities. All research team members completed Collaborative Institutional Training Initiative (CITI) and Health Insurance Portability and Accountability Act (HIPAA) training prior to research activities.

### Enrollment and consent

After confirming eligibility, and per approval by the ethical review entities, staff trained in the protection of human subjects obtained oral consent by reading a consent script aloud and ensuring comprehension by soliciting and answering questions. After both the respondent and a research team member signed the consent, the respondent then completed a paper- or tablet-based survey. Respondents were given a raffle ticket in exchange for their time. At the end of each survey event, staff raffled off 3 health promotion and hygiene items valued at $10-$30 each.

### Data collection and measures

Surveys were collected in 2 rural communities on the Navajo Nation (communities A and B). The research team, including community-based interventionists, developed this survey based on recommendations from Community Advisory Boards (CABs) made up of key stakeholders in both communities. The survey developed for this study is provided as Additional file 1. The 21-question survey explored the following domains: (1) health communication around the transition to womanhood; (2) cultural assets during the transition to womanhood; (3) perceptions of mother-daughter reproductive health communication; and (4) mother-daughter intervention health topics. The survey was piloted with 4 adult AI women at the two study sites before implementation.

**Fig. 1:**
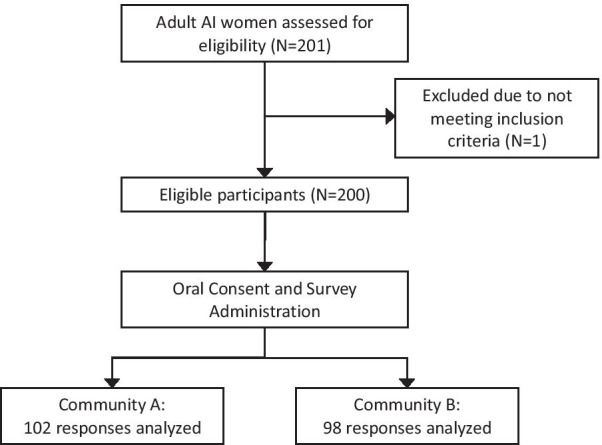
Inclusion and exclusion flow diagram

The self-administered survey was administered as primarily paper-based (N = 175), with a small number administered via tablet (N = 25). Validated measures were not used as the survey was exploratory, AI-culture specific, and formative in nature (Additional file 1). The survey was created in Research Electronic Data Capture (REDCap™) and initially designed for both tablet- and paper-based administration. All surveys were cross-checked with the REDCap™ database for accuracy. The survey was administered to a total of 201 respondents at 6 events between May and December 2018. Of the 201 total respondents, 1 was excluded for not meeting the eligibility criteria, resulting in an analytic sample size of 200 (Fig. 1).

The JHCAIH research team also reviewed existing substance abuse prevention and teen pregnancy prevention curricula and, based on the review, compiled suggested topics to be included in the mother-daughter program. CAB members provided feedback and the resulting 6 topics were presented in the survey to assess Diné female preferences.

### Statistical analysis

Univariate descriptive analyses, including frequencies and percentages, were conducted for each survey question. Chi squared tests for independence were used to test group differences with regard to community and age group. Fisher’s Exact test was used to test for independence if expected cell counts were less than 5. Significance was set at p ≤ 0.05. All analyses were conducted in STATA, version 15.1 (StataCorp LP).

## Results

### Participant demographics

Participants ranged in age from 18 to 82 years, with a mean age of 44 ± 15.5 years. Participant geographic distribution was nearly equal, with 51% (N=102) and 49% (N = 98) living closest to Communities A and B, respectively. Community B had significantly younger respondents (m = 41.5 years vs. 46.5 years, p = 0.03) (Table 1). Women self-identified as mothers (95; 48%), aunts (59; 30%), older sisters (55; 28%), grandmothers (37; 19%), and/or all of the aforementioned (50; 25%). 35% of women identified as married (N = 69), 32% as single (N = 64), 20% indicated they were not married but had a partner (N = 39), 8% were divorced (N = 16), and 5% were widowed (N = 9, Table 1). Differences in marital status were statistically significant by community (Table 1, p = 0.02).

**Table 1:** Demographic characteristics of participants, May - December 2018

Characteristics	N, (%)	Community A	Community B	p-value
Total	200	102	98	0.46
Age group (years)				0.16
< 20	1 (1%)	0	1	
20–29	42 (21%)	14	28	
30–39	44 (22%)	25	19	
40–49	30 (15%)	14	16	
50–59	36 (18%)	21	15	
60–69	28 (14%)	15	13	
70+	9 (5%)	6	3	
Did not answer	10 (5%)	7	3	
Average ± SD	44 ± 15.5	46.5	41.5	**0.03**
Which of the following describes you?^a^				0.52
Mother	95 (48%)	45	50	
Grandmother	37 (19%)	20	17	
Aunt	59 (30%)	28	31	
Older sister	55 (28%)	31	24	
None of the above	3 (2%)	0	3	
All of the above	50 (25%)	26	24	
Marital status				**0.02**
Single	64 (32%)	34	30	
Not married but have partner	39 (20%)	12	27	
Married	69 (35%)	43	26	
Widowed	9 (5%)	3	6	
Divorced	16 (8%)	9	7	
Did not answer	3 (2%)	1	2	

Percentages may not equal 100 due to rounding

Significant p-values at the 0.05 level are in bold

^a^More than one answer allowed

### Health communication around the transition to womanhood

Reflecting on who they admired most as a young girl, 72% of respondents provided 1 response (as requested). Of the single responses (N = 144), 42% (N = 61) indicated their mother, 24% (N = 34) chose their grandmother, 11% (N = 16) chose their older sister, 8% (N = 12) selected their aunt, 7% (N = 10) indicated other, and 6% (N = 8) indicated their father (Table 2). Of the 27% who gave > 1 response (N = 54), the following were chosen as one of their answers: mother (44; 81%), grandmother (31; 57%), aunt (21; 39%), father (20; 37%), older sister (17; 32%), close friends (9; 17%), and other (4; 7%). “Other” write-in responses (N = 14) included male relatives (i.e. brother or grandfather), extended family, teachers, and counselors.

**Table 2:** Health communication and role models during the transition to womanhood

	N (%)	Community A	Community B	p-value
When you were a young girl (8–11 years old), who did you look up to most? (Circle one.)				
One response (N = 144)				0.78
Mother	61 (42.4%)	29	32	
Father	8 (5.6%)	3	5	
Grandmother	34 (23.6%)	15	19	
Older sister	16 (11.1%)	6	10	
Aunt	12 (8.3%)	65	79	
Friends	3 (2.1%)	1	2	
Other	10 (6.9%)	7	3	
Two or more responses (N = 54)^a^				0.60
Mother	44 (81.5%)	27	17	
Father	20 (37.0%)	13	7	
Grandmother	31 (57.4%)	24	7	
Older sister	17 (31.5%)	12	5	
Aunt	21 (38.9%)	15	6	
Friends	9 (16.7%)	5	4	
Other	4 (7.4%)	4	0	
How old were you when someone first spoke to you about your period?	200			0.15
Younger than 8 years old	24 (12.0%)	8	16	
8–9 years old	30 (15.0%)	15	15	
10–11 years old	58 (29.0%)	36	22	
12–13 years old	36 (18.0%)	17	19	
14–15 years old	19 (9.5%)	11	8	
16 years old or older	9 (4.5%)	4	5	
I don’t remember	21 (10.5%)	8	13	
Did not answer	3 (1.5%)	3	0	
Remembering back to when you had your first period, did you feel comfortable talking to your mother about what you were going through?	200			0.77
Yes	120 (60.0%)	58	62	
No	62 (31.0%)	33	29	
Prefer not to answer (or not applicable)	16 (8.0%)	10	6	
Did not answer	2 (1.0%)	1	1	

^a^27% of respondents circled more than 1 answer

Nearly 30% (N = 58) of women indicated they were 10–11 years old when someone first spoke to them about their menstrual period (Table 2). 12% (N = 24) recalled having this conversation at < 8 years old, 15% (N = 30) at 8–9 years old, 18% (N = 36) at 12–13 years old, 9.5% (N = 19) were 14–15 years old, and 4.5% (N = 9) at 16 years or older. Although women in Community A appeared to have this discussion at a slightly younger age, the difference between community responses was not significant (Table 2, p = 0.15).

A majority of women (120; 60%) recalled feeling comfortable talking to their mother around the time of menarche (Table 2). Nearly one-third (62; 31%) were not comfortable talking to their mother around the time of menarche and 8% (N = 16) preferred not to answer the question. Of those who were not comfortable talking to their mother (N = 62), 24% (n = 15) were most comfortable talking to their sister, while others were most comfortable speaking with their grandmother (8; 13%), no one (8; 13%), aunt (5; 8%), non-relative (e.g. dorm aide or friend’s mother, N = 5; 8%), cousin (3; 5%) or male relative (2; 5%).

### Cultural assets during the transition to womanhood

A vast majority of women (172; 86%) felt their Diné culture was a source of strength (Table 3). More than half (101; 51%) reported having a rite of passage event/celebration (Table 3). Of those who had a rite of passage event/celebration, 93% (N = 94) indicated it was a traditional ceremony (i.e. Kinaaldá is the Diné female puberty ceremony). Younger women (< 39 years) reported having a traditional ceremony more frequently than older women (Table 4). Community B had significantly more rite of passage event/celebration responses than Community A (61 vs. 40 p = 0.004). Of those who did not have a rite of passage event (N = 90), 50% (N = 45) wished they did, 29% (N = 26) did not wish they did, 10% (N = 9) preferred not to answer this question, and 8% (N = 7) erroneously answered this question (i.e. the skip pattern was not followed).

**Table 3:** Cultural assets during puberty: results by community, N (%)

	Total	Community A	Community B	p-value
Total	200	102	98	
Do you see your Diné culture as a source of strength?				0.82
Yes	172 (86%)	87 (85%)	85 (87%)	
No	11 (6%)	5 (5%)	6 (6%)	
Prefer not to answer	12 (6%)	7 (7%)	5 (5%)	
Did not answer	5 (3%)	3 (3%)	2 (2%)	
Remembering back to when you had your first period, did you have a rite of passage ceremony or celebration?				**0.004**
Yes	101 (51%)	40 (39%)	61 (62%)	
No	90 (45%)	56 (55%)	34 (35%)	
Prefer not to answer	7 (4%)	4 (4%)	3 (3%)	
Did not answer	2 (1%)	2 (2%)	0 (0%)	
If you did have a rite of passage ceremony/celebration (n = 101), what type did you have (circle all that apply)^a^	101	40	61	0.60
Traditional	94 (93%)	39 (98%)	55 (90%)	
Modern	4 (4%)	0 (%)	4 (7%)	
Church-related	7 (7%)	2 (5%)	5 (8%)	
Other celebration	1 (1%)	1 (2%)	0 (0%)	
If you did not have a rite of passage event (n = 90), do you wish you did?	90	56	34	0.18
Yes	45 (50%)	24 (43%)	21 (62%)	
No	26 (29%)	18 (32%)	8 (24%)	
N/A	7 (8%)	5 (9%)	2 (6%)	
Prefer not to answer	9 (10%)	8 (14%)	1 (3%)	
Did not answer	3 (3%)	1 (2%)	2 (6%)	

Percentages reflect column totals and may not equal 100 due to rounding

Significant p-values at the 0.05 level is in bold

^a^Percentages reflect column totals and do not equal 100 due to allowance of multiple answers

**Table 4:** Cultural assets during puberty: results by age group, N (%)

	Total	Age group (years)							p-value
		< 20	20–29	30–39	40–49	50–59	60–69	70+	
Do you see your Diné culture as a source of strength?	200	1	42	44	30	36	28	9	0.94
Yes	172 (86%)	1 (100%)	33 (79%)	39 (89%)	27 (90%)	31 (86%)	25 (89%)	7 (78%)	
No	11 (6%)	0 (0%)	4 (10%)	1 (2%)	2 (7%)	2 (6%)	1 (4%)	0 (0%)	
Prefer not to answer	12 (6%)	0 (0%)	3 (7%)	3 (7%)	1 (3%)	2 (6%)	2 (7%)	1 (11%)	
Did not answer	5 (3%)	0 (0%)	2 (5%)	1 (2%)	0 (0%)	1 (3%)	0 (0%)	1 (11%)	
Remembering back to when you had your first period, did you have a rite of passage ceremony or celebration?	200	1	42	44	30	36	28	9	0.36
Yes	101 (51%)	1 (100%)	26 (62%)	21 (48%)	12 (40%)	21 (58%)	12 (43%)	3 (33%)	
No	90 (45%)	0 (0%)	16 (38%)	22 (50%)	16 (53%)	12 (33%)	14 (50%)	6 (67%)	
Prefer not to answer	7 (4%)	0 (0%)	0 (0%)	1 (2%)	2 (7%)	2 (6%)	2 (7%)	0 (0%)	
Did not answer	2 (1%)	0 (0%)	0 (0%)	0 (0%)	0 (0%)	1 (3%)	0 (0%)	0 (0%)	
If you did have a rite of passage ceremony/celebration, what type did you have (circle all that apply)^a^	101	1	26	21	12	21	12	3	**0.04**
Traditional	101 (51%)	1 (100%)	25 (96%)	21 (100%)	9 (75%)	19 (90%)	11 (92%)	3 (100%)	
Modern	90 (45%)	0 (0%)	0 (0%)	0 (0%)	0 (0%)	2 (10%)	2 (17%)	0 (0%)	
Church-related	7 (4%)	0 (0%)	2 (8%)	0 (0%)	3 (25%)	2 (10%)	0 (0%)	0 (0%)	
Other celebration	2 (1%)	0 (0%)	0 (0%)	0 (0%)	0 (0%)	0 (0%)	0 (0%)	1 (33%)	
If you did not have a rite of passage event, do you wish you did?	90	0	16	22	16	12	14	6	**0.025**
Yes	45 (50%)	0 (0%)	13 (81%)	13 (59%)	7 (44%)	2 (17%)	6 (43%)	2 (33%)	
No	26 (29%)	0 (0%)	2 (12%)	4 (18%)	6 (38%)	7 (58%)	5 (36%)	1 (17%)	
N/A	7 (8%)	0 (0%)	0 (0%)	1 (5%)	0 (0%)	2 (17%)	3 (21%)	1 (17%)	
Prefer not to answer	9 (10%)	0 (0%)	1 (6%)	4 (18%)	2 (12%)	1 (8%)	0 (0%)	1 (17%)	
Did not answer	3 (3%)	0 (0%)	0 (0%)	0 (0%)	1 (6%)	0 (0%)	0 (0%)	1 (17%)	

Percentages reflect column totals and may not equal 100 due to rounding

Significant p-values at the 0.05 level are in bold

^a^Percentages reflect column totals and do not equal 100 due to allowance of multiple answers

Although rite of passage event/ceremony occurrence generally appears to be more frequent among younger age groups (Table 4), differences by age group were not significant (p = 0.36). Among those who had a rite of passage event/ceremony, traditional event participation was higher among younger women (Table 4). The difference between age group responses was significant (Table 4, p = 0.04). Of those who did not have a rite of passage event but wished they did, 29% were age 20–29 and 30–39 years; 16% were age 40–49 years, 4% were age 50–59 years, and 18% were age 60 or older (Table 4). The difference between age group responses was significant (p = 0.025).

### Perceptions of mother-daughter reproductive health communication

70% (N = 139) of women indicated they would have liked to learn more about puberty, reproductive health, and relationships when they were a teenager (Table 5). The majority of respondents (134; 67%) felt that Diné mothers and grandmothers are able to teach their children and grandchildren about pregnancy, women’s reproductive health, and relationships (Table 5). Of those who responded otherwise (N = 50), 26% (N = 17) reported Diné mothers and grandmothers do not know how to talk about these sensitive topics; 17% (N = 11) reported such topics are “taboo” to talk about; 15% (N = 9) reported Diné mothers and grandmothers do not know enough about these topics to be able to teach them; 12% (N = 8) indicated “Other” (e.g. language barriers) and 8% (N = 12) felt talking about these topics will encourage sexual activity (Table 5). None of these responses differed significantly by community or by age group.

**Table 5:** Perceptions of mother-daughter reproductive health communication

	N (%)	Community A	Community B	P-value
Would you have liked to learn more about puberty, reproductive health (women’s body parts and how babies are made) and relationships when you were a teenager?	200			0.87
Yes	139 (70%)	70	69	
No	33 (16%)	19	14	
I don’t know	24 (12%)	11	13	
Did not answer	4 (2%)	2	2	
Do you feel that Diné mothers/grandmothers are able to teach their children/grandchildren about pregnancy, women’s reproductive health and relationships?	200			0.35
Yes	134 (67%)	70	64	
No	32 (16%)	19	13	
Not sure	24 (12%)	8	16	
Prefer not to answer	5 (2%)	2	3	
Did not answer	5 (2%)	3	2	
If no, why not?^a^	50			0.51
They do not know enough about these topics to teach their children/grandchildren	9 (15%)	4	5	
They do not know how to talk to their children/grandchildren about these topics	17 (26%)	8	9	
These topics are taboo to talk about	11 (17%)	8	3	
Talking about these topics will encourage them to have sex	5 (8%)	4	1	
Other	8 (12%)	5	3	

^a^Denominator based on the number who responded “no”, “not sure”, or “prefer not to answer” to previous question (n = 66)

### Mother-daughter intervention health topics

A vast majority (greater than 95% for each topic) felt that all of the suggested topics should be taught (Table 6). Additional recommended topics were: incorporating Diné cultural teachings and female etiquette; identifying physical, sexual, mental, and emotional abuse; domestic violence and sexual assault prevention; gender-specific developmental differences; suicide prevention; mental health awareness; self-care and personal hygiene; importance of physical activity; how to seek help; goal-setting; planning for the transition to womanhood; bullying prevention; and recognizing abusive behaviors in relationships. Other general suggestions were value-based, including “being happy,” “treating people the way you want to be treated,” and “self-respect.”

**Table 6:** Preference for suggested curriculum topics

Should this be taught in the program?	Yes [N (%)]
Healthy eating and cooking	198 (100%)
Healthy relationships and communication	197 (100%)
STI prevention	185 (95%)
Reproductive health	190 (98%)
Cultural teachings	185 (99%)
Drug/alcohol prevention	190 (99%)

Suggested cultural elements varied, including teaching about K’e (i.e. Diné clan system and identity); language revitalization; traditional gender roles; womanhood teachings; respecting elders; and teachings on the four sacred mountains. Diné womanhood teachings, or cultural etiquette, referenced teachings that girls receive during the Kinaaldá, including dressing modestly, corn meal grinding, running as prayer, and the responsibilities of Diné women.

Approximately 61% of respondents provided at least 1 answer for the teaching methodology part of questions 15 through 21. Of those who answered “yes” to the first part of each question and selected 1 response for preferred teaching methodology, most respondents (50–74%) recommended teaching each topic to girls and caregivers in 1 group. These results should be interpreted with caution as JHCAIH staff observed several respondents having difficulty in understanding the teaching methodology questions 15 through 21.

## Discussion

### Support for an intergenerational, extended family approach

This survey achieved the goal of identifying cultural assets, perspectives, and formative information vital for developing a culturally grounded intergenerational AI mother-daughter program. Survey results confirm that the Diné women surveyed strongly value matrilineal networks, specifically the bond between girls and their mothers, grandmothers, aunts, and older sisters. While the results confirmed a strong mother-daughter bond, survey respondents struggled to select only one source of support during adolescence. Analysis of both single and multiple responses to this question revealed that women admired their mothers the most but also looked up to their grandmothers, aunts, fathers, and older sisters. These findings reinforce a fundamentally Indigenous belief that childrearing is not restricted to the parents but, rather, is a role undertaken by the entire extended family [52]. Thus, these results support the recommendation that mother-daughter interventions also include extended family members who play a supportive role.

### Intervening in early adolescence

With nearly one-third of women recalling their first menstrual discussion at 10 and 11 years old, results indicate this age would be appropriate to begin program implementation. Intervening during this time period is supported by research indicating early adolescence is a time in which caregivers are still very much engaged with youth and is also a critical time for establishing healthy behaviors [6, 53, 54].

### Supporting female caregivers as health educators

Nearly 70% of women felt that Diné mothers and grandmothers are able to teach their children and grandchildren about pregnancy, women’s reproductive health, and relationships. Although affirmative responses by age group were not significantly different, there was a general trend among younger women to respond “yes” to this question. These findings further support the feasibility of interventions that target both mothers (or female caregivers) and daughters as participants. In addition to benefiting directly from health education, the goal is for mothers/caregivers to reinforce key concepts and support their daughters towards healthier behaviors over the long-term.

### Male sources of support during puberty

The presence of male support during the transition to womanhood was also a prominent theme. When given the opportunity to write-in responses, or when “father” was an answer option, several women selected their father, brother, or grandfather as the person they most admired and/or felt comfortable speaking. Other write-in responses, such as cousin, counselor and teacher, did not specify gender but may have also been male figures. This key finding suggests that mother-daughter interventions should include male sources of support. It also reaffirms positive father engagement as “critical to the healthy social, emotional, and academic outcomes of children at all stages of development” [55]. This inclusion can be implemented by recruiting male caregivers and/or by conducting lessons with male sources of support. Reinforcing positive male sources of support during adolescence has been shown to improve children’s self-esteem, lower depression, and reduce substance use, during a critical development phase [56, 57]. Indigenous fatherhood initiatives recommend male- facilitated, group-based support groups [55, 58]. Thus, survey findings also indirectly support the inclusion of male health educators to facilitate AI girls’ health interventions with male sources of support.

### Support for culturally grounded curricula and programming

Survey results strongly support the integration of cultural teachings into AI mother-daughter interventions. Nearly 9 out of 10 survey respondents viewed their culture as a source of strength and felt that cultural teachings should be taught in the proposed girls’ health program. Most importantly, there was an increasing trend of desire for traditional rite of passage ceremonies among younger age groups. Of those who did not have an event, younger age groups (age 20–39) wished they had a rite of passage event significantly more than older women. These trends may be indicative of broader cultural revitalization momentum and further support the implementation of culturally grounded curricula.

Disruption of culture due to government assimilation policies still has a profound impact on Diné women’s transition to womanhood [59, 60]. Some write-in responses revealed that women were in off-reservation boarding schools during menarche and, without their mother, they confided in dorm aides, teachers, sisters, or cousins. For other boarding school survivors, there was no one to talk to about puberty. This disruption in Diné parenting suggests that not all Diné women received reproductive health education. Therefore, mother-daughter interventions should provide fundamental reproductive health education to both mothers and daughters.

In analyzing rite of passage participation by age, participation dropped sharply from the age groups 50–59 years to 40–49 years before increasing among younger groups (Table 4). This period of stagnant ceremonial participation, primarily among women born between 1969 and 1978, coincided with the resurgence of federal assimilation policies [61]. During this period, the federal government focused on assimilating AIs into mainstream society, providing over 31,000 AIs with incentives for relocation to urban areas [62]. It was not until the 1978 American Indian Religious Freedom Act and the Indian Child Welfare Act were passed that AI ceremonies were decriminalized and the forced removal of AI children to boarding schools or non-AI families through adoption ceased [63]. These prominent policy changes in AI history may partially explain why rite of passage participation drastically reduced between 1949 and 1978. A female dyadic approach to revitalizing protective cultural teachings addresses the possibility that older women were unable to experience their own rite of passage ceremony. Mother-daughter interventions, thus, present an opportunity for both mothers and daughters to gain reproductive health knowledge while also promoting cultural protective factors and healing from historical trauma.

### Limitations

The primary limitations were the small sample size of respondents and the specificity of the tribe. However, given the formative nature of the survey, the sample size was sufficient. External validity is also limited due to the population-specific survey and the heterogeneity of AI tribes. With over 550 federally recognized tribes in the U.S., generalization of review findings to all AI tribes is not possible [52]. Findings are limited to the Diné and, potentially, other regional and/or matrilineal tribes.

Despite piloting the survey, survey design errors were discovered during data collection and analysis. Several write-in responses listed male sources of support during early adolescence, which highlighted a survey design bias toward female sources of support. Since the survey did not include male sources of support as answer options, it is possible the impact of male influences was masked or minimized and should be further explored.

Recall and response biases, as well as memory recall error, were also potential limitations [64]. With a mean age of 44 years, many women were asked to reflect on experiences from over 30 years prior. Recall bias and memory recall error due to telescoping (i.e. inaccurately assigning memories from one time period to another) are both potential limitations [64, 65]. Participant response and/or social desirability biases were also potential limitations as the respondents may have felt certain responses were socially desirable or favored by the research team.

## Conclusions

To our knowledge, this is the first survey conducted to better understand Native females’ experience related to culture and reproductive health and to inform development of a culturally aligned AI female intergenerational program. Survey findings highlighted several cultural assets that contribute to AI girls’ transition to womanhood, including: the influence of extended family members during adolescence; supportive male figures and elements of cultural revitalization; as well as the need to address historical trauma from a strengths-based perspective. Lastly, findings support the design of an intergenerational, culturally responsive intervention to prevent substance use and teen pregnancy in Native communities. Results from this survey were used to develop pilot intervention content, such as: health topics, target age group (9–11 years old), expanded caregiver criteria (i.e. older female relatives), lesson delivery (hybrid of group-based and individual dyads), and protective cultural teachings. Future directions include the development of a complementary fatherhood and/or family-based intervention to prevent Native girls’ substance use and teen pregnancy.

## Supplementary Information

**Additional file 1.** Women's Health Survey.

## Data Availability

Data sharing is not applicable as data is owned by a sovereign tribal nation.

## Abbreviations

AI: American Indian
AIAN: American Indian and Alaska Native
CAB: Community Advisory Board
CITI: Collaborative Institutional Training Initiative
HIPAA: Health Insurance Portability and Accountability Act
JHCAIH: Johns Hopkins Center for American Indian Health
REDCap: Research Electronic Data Capture
