# Supplementary material for: Diné (Navajo) female perspectives on mother–daughter communication and cultural assets around the transition to womanhood: a cross-sectional survey
Source: BMC Womens Health. 2021 Sep 25;21:341. doi: 10.1186/s12905-021-01473-4 (PMC8466980; doi:10.1186/s12905-021-01473-4)
Supplement: Supplementary file 1 — Additional file 1. Women's Health Survey. [file 12905_2021_1473_MOESM1_ESM.docx]

Women’s Health Questionnaire

The Johns Hopkins Center for American Indian Health (JHCAIH) is developing a program to promote Native girls’ health. This questionnaire will ask you about reproductive health and your view on rite of passage ceremonies/celebrations and will help us make an effective program for young women. The survey will take approximately 5 minutes. Thank you for your time.

1. How old are you? ___________
2. Which of the following describes you? Please select all that apply:
   1. Mother
   2. Grandmother
   3. Aunt
   4. Older Sister
   5. None of the above
   6. All of the above
3. Please select which tribal community that you live closest to:
   1. Community A
   2. Community B
4. What is your marital status?
   1. Single
   2. Not married but have partner
   3. Married
   4. Widowed
   5. Divorced
5. Do you see your Navajo culture as a source of strength?
   1. Yes
   2. No
   3. Prefer not to answer
6. When you were a young girl (8-11 years old), who did you look up to most (**Circle one**):
   1. Mother
   2. Father
   3. Grandmother
   4. Older Sister
   5. Aunt
   6. Friends
   7. Other (Please list: _________________________)
   8. Prefer not to answer
7. How old were you when someone first spoke to you about your period?
   1. Younger than 8 years old
   2. 8 – 9 years old
   3. 10 – 11 years old
   4. 12 – 13 years old
   5. 14 – 15 years old
   6. 16 years old or older
   7. I don’t remember
8. Remembering back to when you had your first period, did you feel comfortable talking to your mother about what you were going through?
   1. Yes
   2. No **🡪** Who did you feel most comfortable speaking with: __________________________
   3. Prefer not to answer (or not applicable)
9. Remembering back to when you had your first period, did you have a rite of passage ceremony or celebration?
   1. Yes
   2. No **🡪 Skip to Q11**
   3. Prefer not to answer
10. If you **did** have a rite of passage ceremony/celebration, what type did you have (**circle all that apply**):
    1. Traditional (example: Kinaaldá, traditional initiation, or other ceremony)
    2. Modern (example: informal family celebration)
    3. Church-related
    4. Other (please describe in a few words): _________________________________
    5. Prefer not to answer
11. If you **did not** have a rite of passage ceremony/celebration, do you wish you did?
    1. Yes
    2. No
    3. Not applicable (I did have a ceremony/celebration)
    4. Prefer not to answer
12. Would you have liked to learn more about puberty, reproductive health (women’s body parts and how babies are made) and relationships when you were a teenager?

a) Yes

b) No

c) I don’t know

1. Do you feel that Navajo mothers/grandmothers are able to teach their children/grandchildren about pregnancy, women’s reproductive health and relationships?
   1. Yes **🡪 Skip to Q15**
   2. No
   3. Not sure
   4. Prefer not to answer
2. If no, why not?
   1. They **do not know enough** about these topics to teach their children/grandchildren
   2. They **do not know how** to talk to their children/grandchildren about these topics
   3. These **topics are taboo** to talk about
   4. **Talking about** **these topics will encourage them** to have sex
   5. Other: _____________________________________________________

**We are developing a program to improve the health of young girl’s (ages 8-11) (a female caregiver such as her mother, aunt, grandmother or other female relative will also participate). The questions below ask about this program.**

|  | Should this be taught in the program? | To whom should it be taught and how?  **Please Circle all that apply** | | | |
| --- | --- | --- | --- | --- | --- |
| 1. Healthy eating and cooking (nutrition education) | Yes No | Just teach a group of female caregivers | Just teach a group of girls | Teach all girls and female caregivers in 1 group | Individual pairs (1 girl and her female caregiver) |
| 1. Healthy relationships and communication (with friends/family) | Yes No | Just teach a group of female caregivers | Just teach a group of girls | Teach all girls and female caregivers in 1 group | Individual pairs (1 girl and her female caregiver) |
| 1. Sexually Transmitted Infection (STI) prevention | Yes No | Just teach a group of female caregivers | Just teach a group of girls | Teach all girls and female caregivers in 1 group | Individual pairs (1 girl and her female caregiver) |
| 1. Reproductive health (e.g. women’s body parts how babies are made) | Yes No | Just teach a group of female caregivers | Just teach a group of girls | Teach all girls and female caregivers in 1 group | Individual pairs (1 girl and her female caregiver) |
| 1. Cultural teachings (on healthy womanhood) | Yes No | Just teach a group of female caregivers | Just teach a group of girls | Teach all girls and female caregivers in 1 group | Individual pairs (1 girl and her female caregiver) |
| 1. Drug/alcohol prevention | Yes No | Just teach a group of female caregivers | Just teach a group of girls | Teach all girls and female caregivers in 1 group | Individual pairs (1 girl and her female caregiver) |
| 1. Other topics (Please list below): | | Just teach a group of female caregivers | Just teach a group of girls | Teach all girls and female caregivers in 1 group | Individual pairs (1 girl and her female caregiver) |

***Thank you for your time!***
